# Exosomal PSMD1 Derived From Dental Pulp Mesenchymal Stem Cells Promotes Microglial M2 Polarization to Alleviate Spinal Cord Injury

**DOI:** 10.1002/cns.71133

**Published:** 2026-09-02

**Authors:** Xiaohui Xing, Yuan Hao, Guanghui Zhang, Xiaoyang Xing, Hongfei Jia, Chenlong Liu, Xin Wang, Tao Li, Xueyuan Li, Yilei Xiao

**Affiliations:** ^1^ Department of Neurosurgery Shandong First Medical University Affiliated Liaocheng People's Hospital Liaocheng Shandong China; ^2^ Department of Liaocheng Key Laboratory of Brain Science and Neurological Disorders Innovation Technology Shandong First Medical University Affiliated Liaocheng People's Hospital Liaocheng Shandong China; ^3^ Department of Neurosurgery The Second Hospital of Shandong University Jinan Shandong China; ^4^ Department of Neurosurgery Brigham and Women's Hospital, Harvard Medical School Boston Massachusetts USA; ^5^ Department of Pathology Liaocheng People's Hospital Liaocheng Shandong China; ^6^ Department of Clinical Laboratory Liaocheng Maternal and Child Health Hospital Liaocheng Shandong China; ^7^ Department of Spinal Surgery Shandong Provincial Hospital Affiliated to Shandong First Medical University Jinan Shandong China

**Keywords:** BV2 microglia, dental pulp mesenchymal stem cells, exosomes, polarization, PSMD1, spinal cord injury

## Abstract

**Background:**

Exosomes derived from mesenchymal stem cells (MSCs) and their cargo contribute to the protective properties of MSCs in spinal cord injury (SCI). This study aimed to explore the role of Proteasome 26S subunit non‐ATPase 1 (PSMD1), delivered via exosomes derived from dental pulp mesenchymal stem cells (DPMSCs‐Exo), in SCI repair.

**Methods:**

Proteomic profiling was conducted to identify proteins enriched in DPMSCs‐Exo. M1/M2 microglial polarization was assessed using flow cytometry, immunofluorescence, and western blotting. The apoptotic rates of BV2 microglia and NE‐4C neural stem cells were measured by flow cytometry, whereas cell viability was determined using the CCK‐8 assay. Western blotting was performed to analyze proteins involved in autophagy and apoptosis. Pro‐inflammatory cytokine levels were quantified using ELISA. Additionally, a mouse SCI model treated with DPMSCs‐Exo was established to assess therapeutic effects in vivo.

**Results:**

DPMSCs‐Exo enriched with PSMD1 suppressed M1‐associated inflammatory activation, promoted M2 microglial polarization, and suppressed inflammation in BV2 cells following LPS exposure by activating the JAK2/STAT3 pathway. The shift toward an anti‐inflammatory microglial phenotype indirectly enhanced survival, reduced apoptosis, and inhibited autophagy in NE‐4C neural stem cells. In SCI mice, exosomal PSMD1 improved locomotor recovery, promoted spinal tissue regeneration at the injury site, reduced inflammation and astrocyte activation, and increased M2 microglial polarization.

**Conclusions:**

DPMSCs‐Exo carrying PSMD1 suppressed M1‐type inflammatory responses while promoting M2 microglial polarization, thereby attenuating inflammation and astrocyte activation and facilitating SCI repair. These findings suggest that DPMSCs‐Exo may be a promising therapeutic candidate for the treatment of SCI.

## Graphical abstract

DPMSCs‐derived exosomal PSMD1 stimulates M2 polarization through the JAK2/STAT3 pathway. DPMSCs‐derived exosomal PSMD1 indirectly acts on NE‐4C cells. DPMSCs‐derived exosomal PSMD1 ameliorates SCI in a mouse model.

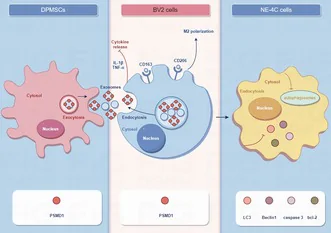

**Abbreviations**
BBB: Basso, Beattie, and Bresnahan
BSA: bovine serum albumin
CSF: cerebrospinal fluid
DPMSCs‐Exo: exosomes derived from dental pulp mesenchymal stem cells
FBS: fetal bovine serum
GO: Gene Ontology
KEGG: Kyoto Encyclopedia of Genes and Genomes
MSCs: mesenchymal stem cells
NGF: nerve growth factor
NT‐3: neurotrophin‐3
NTA: nanoparticle tracking analysis
OE‐NC: negative control vectors
OE‐PSMD1: PSMD1 overexpression vectors
PBS: phosphate‐buffered saline
PSMD1: proteasome 26S subunit non‐ATPase 1
SCI: spinal cord injury
TEM: transmission electron microscopy
UPS: ubiquitin‐proteasome system

## 1 Background

Spinal cord injury (SCI) is a devastating neurological condition resulting in partial or complete loss of sensory, autonomic, and motor functions, placing a significant burden on affected individuals and healthcare systems worldwide [1]. Globally, an estimated 250–500 thousand new cases of SCI occur each year, and many patients experience lifelong disability and a substantially diminished quality of life [2]. In addition to the initial mechanical trauma, SCI triggers a series of secondary injury processes, including excessive inflammation, glial activation, neuronal apoptosis, edema, and oxidative stress, all of which aggravate tissue damage and impede recovery [3]. The subsequent infiltration of inflammatory cells and activation of astrocytes contribute to fibrotic glial scar formation, which constitutes a major barrier to axonal regeneration and neural circuit reconstruction [4]. Although advances in clinical management, such as spinal stabilization, decompression, and fracture reduction, have improved acute care, these interventions primarily focus on limiting secondary damage rather than directly promoting neural regeneration [5]. Emerging strategies (e.g., optogenetic approaches) have shown therapeutic potential in experimental models but remain far from clinical implementation [6]. Therefore, there is a critical need for effective therapies that can modulate the post‐injury microenvironment, promote neural repair, and facilitate functional recovery following SCI.

Mesenchymal stem cells (MSCs) are promising therapeutic candidates for SCI due to their potent immunomodulatory and regenerative properties [7]. MSCs derived from various tissues, including umbilical cord, bone marrow, and adipose tissue, have been widely studied in preclinical SCI models and early‐stage clinical trials [8, 9, 10]. Accumulating evidence indicates that the therapeutic benefits of MSCs are predominantly mediated through paracrine mechanisms that modulate the inflammatory microenvironment, inhibit apoptosis, promote angiogenesis, and support endogenous neural repair [11, 12]. Among these paracrine factors, MSC‐derived exosomes, nanosized extracellular vesicles enriched with bioactive nucleic acids, lipids, and proteins, have garnered increasing attention as critical mediators of MSC function [13]. Compared with cell‐based approaches, exosome‐based therapies provide several advantages, including low immunogenicity, high stability, efficient penetration of the blood‐spinal cord barrier, and reduced risks related to cell transplantation, such as vascular occlusion or tumorigenicity [14]. Consistently, MSC‐derived exosomes can attenuate neuroinflammation, support axonal regeneration, and improve functional recovery in experimental SCI models, highlighting their translational potential [15].

Dental pulp MSCs (DPMSCs) are isolated from discarded teeth through minimally invasive procedures and exhibit multilineage differentiation potential comparable to that of other MSCs. They express various immunomodulatory and neurotrophic factors, facilitating neuroregeneration and angiogenesis [16]. Importantly, DPMSCs share a neural crest origin with components of the central nervous system, suggesting an inherent suitability for neural repair [17]. Luzuriaga et al. [18] reported that, under specific induction conditions, DPMSCs can differentiate into neuron‐like cells while expressing neural stem cell markers; these findings were later supported by Darvishi et al. [19]. In vivo, Qian et al. [20] demonstrated that DPMSCs delivered via microsphere scaffolds promoted neurogenic differentiation and functional recovery in a rat SCI model [20]. Similarly, Liu et al. showed that conditioned medium from DPMSCs partially restored sensory and motor function in SCI rats [21]. Moreover, DPMSC‐derived exosomes regulate macrophage polarization and suppress pro‐inflammatory cytokine production, thereby attenuating SCI progression [22]. Despite these advances, the specific exosomal proteins from DPMSCs responsible for these reparative effects remain largely unidentified.

Dysregulation of the ubiquitin‐proteasome system (UPS) has been increasingly recognized as a critical contributor to neurodegeneration and neuroinflammation [23]. As the principal intracellular machinery responsible for protein quality control, the UPS maintains cellular homeostasis by degrading damaged, misfolded, and regulatory proteins involved in cell survival, immune responses, and signal transduction [24]. Impaired proteasomal function can lead to the accumulation of toxic protein aggregates, aberrant inflammatory activation, and neuronal dysfunction. These processes are implicated in a variety of neurological disorders [25]. Among the components of the UPS, proteasome 26S subunit non‐ATPase 1 (PSMD1) is a core structural subunit of the 19S regulatory particle that serves as a scaffold for the assembly of additional proteasomal subunits, thereby playing a central role in maintaining protein homeostasis [26]. PSMD1 has a well‐established role in cancer biology [27], where it regulates cell survival and apoptosis by proteasome‐mediated degradation of substrates (e.g., p53 [28], NOXA [29]). Emerging evidence suggests that proteasomal activity is closely linked to the regulation of innate immune responses and inflammatory signaling [30]. The UPS participates in the activation of multiple inflammatory pathways, including NF‐κB‐ and STAT3‐related signaling networks. It has been implicated in the regulation of macrophage and microglial phenotypic polarization [31]. In addition, PSMD1 is associated with altered neuroimmune homeostasis and the progression of some neurodegenerative diseases such as Parkinson's and Alzheimer's disease [32, 33]. However, whether PSMD1 contributes to neuroinflammation and neural repair in SCI remains unknown.

Recent studies have identified the JAK2/STAT3 pathway as a key regulator of microglial polarization and neuroinflammatory responses [34, 35]. Activation of JAK2/STAT3 signaling promotes the expression of anti‐inflammatory and tissue‐repair‐associated genes while suppressing excessive pro‐inflammatory responses, thereby contributing to functional recovery after central nervous system injury [36]. Accumulating evidence indicates that exosome‐mediated modulation of JAK2/STAT3 signaling can influence macrophage and microglial polarization in SCI and other neurological disorders [37]. Given the close interplay between proteasomal function, inflammatory signaling, and microglial activation, we hypothesized that PSMD1, if enriched in DPMSC‐derived exosomes, could be transferred to microglia to modulate their inflammatory phenotype through the JAK2/STAT3 pathway, thereby attenuating neuroinflammation and promoting neural repair following SCI. Therefore, this study was designed to explore whether DPMSC‐derived exosomal PSMD1 enhances neural stem cell survival and ultimately facilitates functional recovery following SCI, using both in vitro and in vivo models.

## 2 Materials and Methods

### 2.1 Animals, Cells, and Reagents

Adult female BALB/c mice (26–30 g) were purchased from Vital River Laboratories (Beijing, China). Human UCMSCs (cat. no. CP‐CL11), human BMMSCs (cat. no. CP‐H166Y), mouse microglia BV2 cells (cat. no. CL‐0493), and mouse neural stem cells NE‐4C (cat. no. CP‐M139), along with their respective growth media, were obtained from Procell Life Science and Technology (Wuhan, China). Human DPMSCs (cat. no. G02011) were obtained from Bohui Biotechnology (Guangzhou, China), and the culture medium was purchased from Haixing Biosciences (Suzhou, China). All cells tested negative for mycoplasma contamination.

The GENESEED Exosome Isolation Reagent Kit was provided by Geneseed Biology (Guangzhou, China), and the ExoQuick Exosome Precipitation Kit was obtained from System Biosciences (Mountain View, CA, USA). Stattic, a STAT3 inhibitor, was purchased from MedChemExpress (Shanghai, China). Lipopolysaccharide (LPS), PKH26 dye, and Alexa Fluor 488‐phalloidin were purchased from Sigma‐Aldrich (St. Louis, MO, USA). Hoechst 33,342 and primary antibodies against iNOS, CD206, CD163, CD68, GFP, PSMD1, LC3II, LC3I, Bcl‐2, caspase‐3, cleaved caspases‐3, Bax, nerve growth factor (NGF), neurotrophin‐3 (NT‐3), p‐JAK2, JAK2, p‐STAT3, STAT3, p‐STAT6, STAT6, p‐ERK, ERK, p‐CEBPβ, CEBPβ, NeuN, and GAPDH, as well as the corresponding HRP‐ or fluorophore‐conjugated secondary antibodies, were purchased from Proteintech (Manchester, UK). Primary antibodies against CD9, Beclin‐1, GFAP, and vimentin were obtained from Cell Signaling Technology (Danvers, MA, USA). Antibodies against CD81, TSG101, and Ki67 were obtained from Abcam (Cambridge, UK). The pcDNA3.1(+)‐eGFP plasmid (Addgene plasmid #129020) was a gift from Jeremy Wilusz.

ELISA kits for mouse IL‐1β, CXCL12, and TNF‐α, as well as the hematoxylin and eosin (H&E) staining kit, BCA protein assay kit, CCK‐8 kit, Annexin V–FITC apoptosis detection kit, bovine serum albumin (BSA), fetal bovine serum (FBS), and phosphate‐buffered saline (PBS), were obtained from Beyotime (Shanghai, China). The ECL basic kit was purchased from ABclonal Technology (Wuhan, China). The peptide quantification kit was obtained from Thermo Fisher Scientific (Waltham, MA, USA). PSMD1 overexpression vectors (OE‐PSMD1) and the corresponding negative control vectors (OE‐NC) were synthesized by Ribo Biotech (Guangzhou, China). Lipofectamine 3000 and DiR dye were obtained from Invitrogen (Carlsbad, CA, USA).

### 2.2 Patient Samples

Six patients with spinal cord injury (≥ 24 h after onset; aged ≥ 18 years) were recruited from our hospital. Patients with head trauma or other non‐SCI‐related injuries were excluded. Six age‐matched healthy individuals were selected as the control group. Individuals with inflammatory, neurological, or metabolic disorders were excluded.

Peripheral blood samples were collected within 48 h. Plasma was centrifuged at 4°C for 10 min at 3000 × g within 4 h of collection. The supernatant was further centrifuged at 4°C for 10 min at 16,000 × g, and plasma samples were stored at −80°C for further analysis.

### 2.3 Cell Culture and Transfection

UCMSCs, BMMSCs, BV2, NE‐4C, and DPMSCs were cultured in their respective media supplemented with 10% FBS in a 5% CO_2_ incubator at 37°C. To mimic SCI in vitro, BV2 or NE‐4C cells were stimulated with 1 μg/mL LPS for 48 h. To evaluate the direct functional effect of PSMD1 on BV2 microglial polarization, BV2 cells or DPMSCs were transfected with OE‐PSMD1 or OE‐NC plasmids using Lipofectamine 3000. For PSMD1 knockdown experiments, DPMSCs were transfected with two small interfering RNAs (siRNAs) targeting PSMD1 (si‐PSMD1‐1 and si‐PSMD1‐2) or a negative control siRNA (si‐NC) using Lipofectamine 3000. The sequences were as follows: si‐PSMD1‐1, sense 5′‐UUUGUGUAGUGCAAAUUCCUU‐3′ and antisense 5′‐GGAAUUUGCACUACACAAAUU‐3′; si‐PSMD1‐2, sense 5′‐AUUUGUGUAGUGCAAAUUCCU‐3′ and antisense 5′‐GAAUUUGCACUACACAAAUUG‐3′. Functional assays were performed 48 h after transfection.

### 2.4 Exosome Isolation and Characterization

MSCs were maintained in culture medium (exosome‐free) for 72 h at 37°C with 5% CO_2_. To remove debris, supernatants were collected and centrifuged, and exosomes were isolated using the GENESEED Exosome Isolation Reagent Kit. The obtained mixture was incubated at 4°C for 30 min, then centrifuged at 2000 × g for 30 min. Plasma and cerebrospinal fluid (CSF) samples from SCI patients (SCI‐Exo) and healthy controls (Normal‐Exo) were processed using the ExoQuick Exosome Precipitation Kit. Isolated exosomes were resuspended in PBS for subsequent analyses.

Transmission electron microscopy (TEM) was used to examine exosomal morphology and autophagosomes. Nanoparticle tracking analysis (NTA) was used to assess exosome size and distribution. Western blotting was conducted to evaluate the expression of exosomal markers CD9, CD81, and TSG101.

### 2.5 Exosome Uptake Assay

Exosomes derived from UCMSCs (UCMSCs‐Exo), BMMSCs (BMMSCs‐Exo), or DPMSCs (DPMSCs‐Exo) were labeled with PKH26 at 37°C for 30 min and then added to BV2 cells. After 6 h, cells were fixed with 4% paraformaldehyde for 30 min and then incubated with Alexa Fluor 488‐phalloidin for another 30 min. The nucleus was counterstained with Hoechst 33,342 for 10 min. Additionally, a DPMSC model expressing PSMD1‐GFP fusion protein was established as previously described [38]. Confocal microscopy (Olympus, Tokyo, Japan) was used to visualize PKH26‐labeled exosomes and GFP‐positive cells.

### 2.6 Co‐Culture System

To determine whether exosomal PSMD1 influenced neural stem cells directly or indirectly via BV2 cells, a Transwell co‐culture system was used. Briefly, BV2 cells and DPMSCs‐Exo containing PSMD1 were seeded in the upper chamber (donor compartment), while LPS‐treated NE‐4C cells were plated in the lower chamber (recipient compartment). After 24 h of incubation, NE‐4C cells were collected for subsequent assays.

### 2.7 Cell Viability and Apoptosis Measurement

Cell viability in BV2 and NE‐4C cells was evaluated using the CCK‐8 assay. Cells were plated into 96‐well plates (3 × 10^3^ cells/mL) and cultured for 0–72 h. CCK‐8 solution (15 μL) was added to each well, and the plate was incubated for 2 h. A microplate reader (Bio‐Rad Laboratories, Hercules, CA, USA) was used to measure the absorbance.

Apoptosis was analyzed using the Annexin V‐FITC apoptosis detection kit. BV2 or NE‐4C cells (2 × 10^5^) were stained with Annexin V and PI (10 μL) for 30 min in a light‐proof room. The apoptotic rate was determined using a FACSCalibur flow cytometer (BD Biosciences, San Jose, CA, USA).

### 2.8 Proteomics Analysis

Based on the effects of UCMSCs‐Exo, BMMSCs‐Exo, and DPMSCs‐Exo on the viability and apoptosis of LPS‐induced BV2 cells, DPMSCs‐Exo was selected for proteomic profiling. Proteins were extracted using protease inhibitor‐containing RIPA buffer and quantified using the BCA assay. After trypsin digestion, peptides were dried under vacuum, redissolved in 0.1% trifluoroacetic acid, desalted through HLB columns, and dried again. Peptide concentrations were measured using the peptide quantification kit.

Peptides were resuspended in a spectrometry loading buffer (2% acetonitrile with 0.1% formic acid) containing iRT calibration peptides and analyzed using an EASY‐nLC system coupled to an Orbitrap Eclipse Tribrid mass spectrometer (Thermo Fisher Scientific). Data were obtained over an m/z range of 350–1500 with an ion mobility range of 0.57–1.47 Vs·cm^−2^. Both the ramp and accumulation time were set to 100 ms. Each acquisition cycle consisted of one MS scan followed by 10 PASEF MS/MS scans. Dynamic exclusion was enabled after 0.4 min.

The proteomic data were used for bioinformatic analysis using the Majorbio Cloud Platform (https://cloud.majorbio.com). Pathway enrichment analysis was performed using the Kyoto Encyclopedia of Genes and Genomes (KEGG) database, while functional annotation of identified proteins was achieved using Gene Ontology (GO) (http://geneontology.org/). Protein–protein interactions were predicted using the STRING database (version 10.0), and the Cytoscape software (version 3.7.1) was used to visualize the interaction network.

### 2.9 SCI Animal Model

Mice were randomly divided into four groups (*n* = 7 per group) after one week of acclimatization: Control + PBS, Model + PBS, Model + DPMSCs‐OE‐NC‐Exo, and Model + DPMSCs‐OE‐PSMD1‐Exo. SCI was induced as previously described [39] with modifications. Mice were anesthetized via [39] intraperitoneal injection of 50 mg/kg pentobarbital sodium, and a dorsal midline incision was made. The paraspinal muscles were separated, a laminectomy was performed at the T10 level, and a contusion injury was induced using an impactor. Control mice received no surgical intervention.

Two hours post‐injury, mice in the treatment groups received intramuscular injections of 100 μL of DiR‐labeled exosomes at the injury site, while control animals received PBS. NIRF imaging was performed using an IVIS Lumina S5 System (Revvity, Shanghai, China). Locomotor recovery was evaluated using the Basso, Beattie, and Bresnahan (BBB) scoring system one week after SCI.

All animals were euthanized via intraperitoneal injection of 200 mg/kg pentobarbital sodium. Blood samples (300 μL) were collected via cardiac puncture, and serum was isolated. Spinal cord tissues were harvested for subsequent analyses. Outcome assessments were performed in a blinded manner.

### 2.10 Histopathologic Examination

Spinal cord tissues were fixed, dehydrated, embedded in paraffin, and sectioned into 5‐μm‐thick slices. Following dewaxing and rehydration, H&E staining was performed. Microscopic analysis was carried out using a light microscope (BX53, Olympus, Tokyo, Japan).

### 2.11 Immunohistochemistry

Following deparaffinization and rehydration, paraffin‐embedded spinal cord sections (5 μm thick) were subjected to antigen retrieval in 10 mM citrate buffer (pH 6.0). Sections were incubated in 3% hydrogen peroxide for 10 min, followed by overnight incubation at 4°C with an anti‐PSMD1 antibody (1:200). After washing, sections were incubated with an HRP‐conjugated secondary antibody for 50 min at 37°C. Visualization was performed using DAB, followed by counterstaining with hematoxylin for 1 min. Images were captured using Image‐Pro Plus software.

### 2.12 Ki67/NeuN Immunofluorescence Staining

After antigen retrieval, spinal cord tissue sections were blocked with goat serum and incubated overnight with primary antibodies against Ki67 (1:500) and NeuN (1:500). A FITC‐labeled secondary antibody (1:500) was then applied, followed by nuclear staining with DAPI. Fluorescence signals were detected using an Olympus fluorescence microscope.

### 2.13 Western Blotting

Protein samples were separated by SDS‐PAGE and transferred onto PVDF membranes. After blocking with 5% BSA at room temperature, the membranes were incubated overnight at 4°C with primary antibodies against CD9 (1:1500), CD81 (1:1500), TSG101 (1:1500), CD206 (1:1000), CD163 (1:1000), PSMD1 (1:1000), GFP (1:1000), LC3II (1:1000), LC3I (1:1000), Beclin1 (1:1000), caspase‐3 (1:1000), Bcl‐2 (1:1000), GFAP (1:1000), vimentin (1:1000), Cleaved caspases‐3 (1:1000), Bax (1:1000), NGF (1:1000), NT‐3 (1:1000), p‐JAK2 (1:1000), JAK2 (1:1000), p‐STAT3 (1:1000), STAT3 (1:1000), p‐STAT6 (1:1000), STAT6 (1:1000), p‐ERK (1:1000), ERK (1:1000), p‐CEBPβ (1:1000), CEBPβ (1:1000), and GAPDH (1:1500). After washing with Tris‐buffered saline containing Tween‐20, the membranes were incubated with an HRP‐conjugated secondary antibody (1:3000) for 1 h at room temperature. GAPDH was used as the loading control. Signals were detected using the ECL basic kit and visualized with a Gel‐Pro Analyzer (version 4.0, USA).

### 2.14 Assessment of M1/M2 Polarization

For immunofluorescence staining, BV2 cells were fixed in 4% paraformaldehyde for 10 min, blocked with 1% BSA, and incubated with primary antibodies against CD68 (1:100), CD163 (1:100), and CD206 (1:100) overnight at 4°C. Spinal cord tissue sections were incubated with antibodies against CD68 (1:100), iNOS (1:100), and CD206 (1:100). Following incubation with Alexa Fluor 488‐conjugated secondary antibodies (1:400) at room temperature for 1 h, nuclei were counterstained with DAPI and imaged using confocal microscopy.

Flow cytometry was also employed to assess M1/M2 polarization. BV2 cells were fixed with 4% paraformaldehyde at room temperature for 10 min and then washed with PBS. A 1% BSA/PBS solution was applied to block nonspecific binding. Then, cells were incubated with primary antibodies against CD68 (1:200), iNOS (1:200), CD163 (1:100), and CD206 (1:400) for 1 h, followed by incubation with Alexa Fluor 488‐ or 555‐conjugated secondary antibodies (1:1000) for an additional hour. Nuclei were counterstained with Hoechst 33342. After final PBS washes, samples were analyzed using a FACSCalibur flow cytometer.

### 2.15 Elisa

Levels of IL‐1β, CXCL12, and TNF‐α in BV2 cells or mouse serum were quantified using commercial ELISA kits.

### 2.16 Statistical Analysis

All in vitro experiments were performed in triplicate and independently repeated three times. In vivo experiments included seven mice per group. Statistical analysis was performed using SPSS 23.0 (SPSS Inc., Chicago, IL, USA). Comparisons among multiple groups were conducted using one‐way ANOVA followed by Tukey's multiple‐comparisons post hoc test. Comparisons between the two groups were performed using Student's *t*‐test. Statistical significance was determined when *p*‐values were less than 0.05.

## 3 Results

### 3.1 Comparison of the Effects of UCMSCs‐Exo, BMMSCs‐Exo, and DPMSCs‐Exo on LPS‐Induced BV2 Cells

**FIGURE 1:**
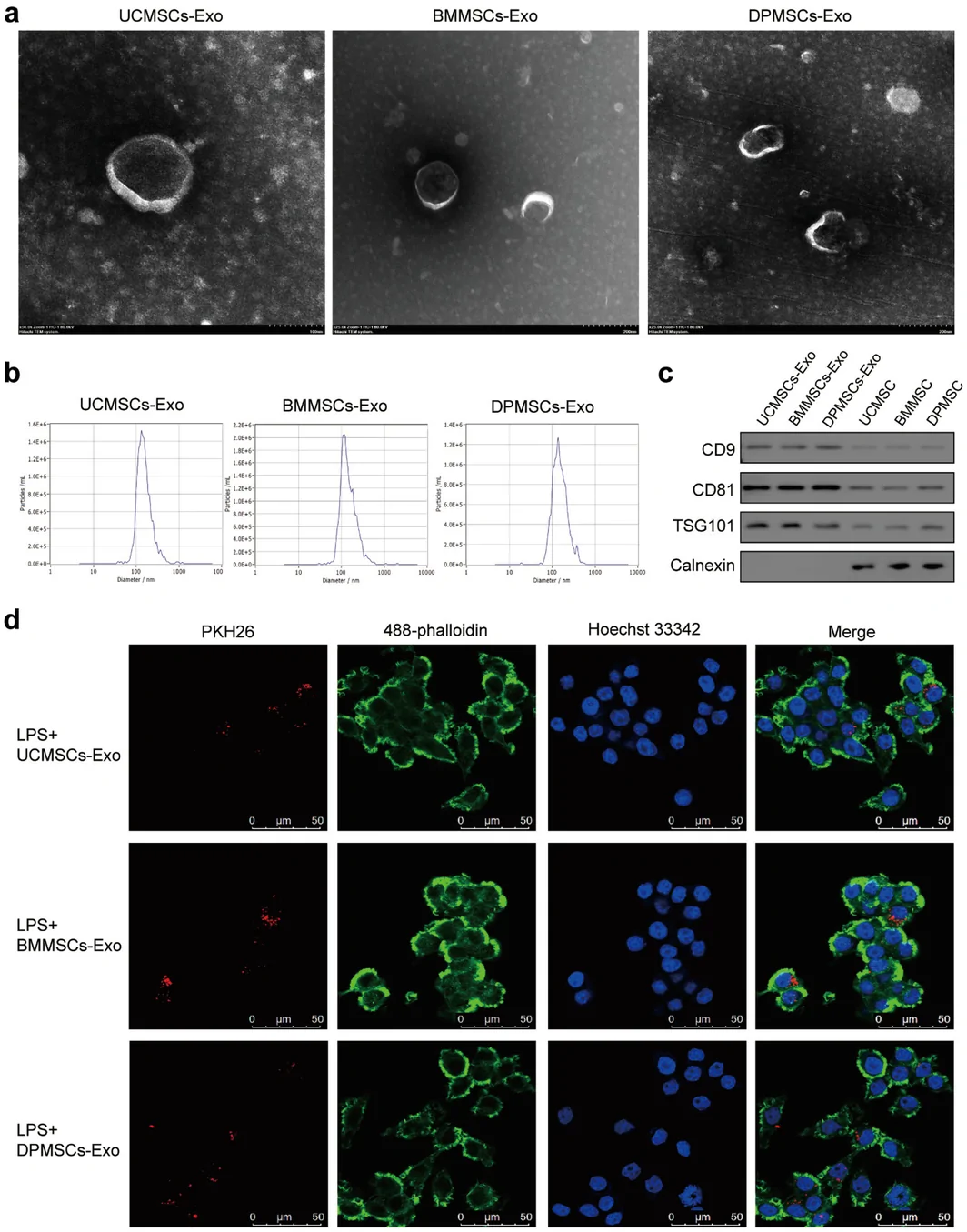
Identification of UCMSCs‐Exo, BMMSCs‐Exo, and DPMSCs‐Exo. (a) The morphology of exosomes isolated from UCMSCs (UCMSCs‐Exo), BMMSCs (BMMSCs‐Exo), and DPMSCs (DPMSCs‐Exo) was visualized by TEM. Scale bar = 200 μm. (b) The size distribution and particle concentration of exosomes were assessed using nanoparticle tracking analysis. (c) Western blot analysis of exosome‐positive markers CD9, CD81, and TSG101, and the negative marker Calnexin in parental cells (UCMSCs, BMMSCs, and DPMSCs) and corresponding exosomes (UCMSCs‐Exo, BMMSCs‐Exo, and DPMSCs‐Exo). (d) To evaluate internalization of UCMSCs‐Exo, BMMSCs‐Exo, and DPMSCs‐Exo by LPS‐induced BV2 cells, exosomes were labeled with PKH26 and then co‐cultured with LPS‐induced BV2 cells.

TEM and NTA were used to characterize UCMSCs‐Exo, BMMSCs‐Exo, and DPMSCs‐Exo. As shown in Figure 1a,b, the isolated vesicles exhibited typical exosomal features, including an average diameter of approximately 100 nm and a bilayer membrane structure. Western blot analysis demonstrated that the exosomal positive markers CD9, CD81, and TSG101 were enriched in all exosome preparations, whereas the endoplasmic reticulum‐associated negative marker Calnexin was absent (Figure 1c), confirming the purity of the isolated exosomes.

To evaluate whether these exosomes could be internalized by LPS‐activated BV2 cells, exosomes were labeled with PKH26 and co‐cultured with BV2 cells. All three exosome types were efficiently internalized (Figure 1d).

**FIGURE 2:**
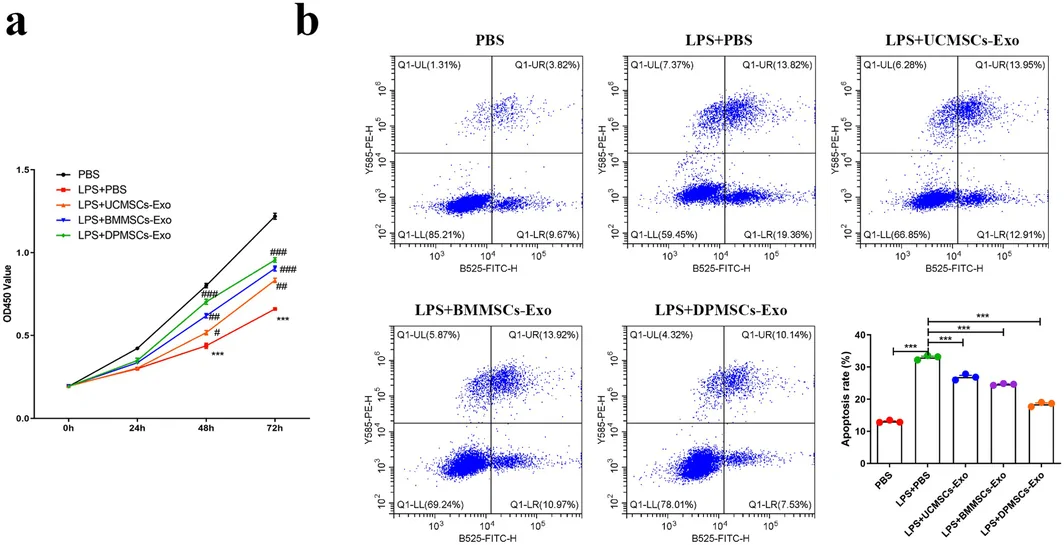
Comparison of the effects of UCMSCs‐Exo, BMMSCs‐Exo, and DPMSCs‐Exo on LPS‐induced BV2 cells. Following uptake of exosomes, the (a) viability and (b) apoptosis of LPS‐induced BV2 cells were measured using the CCK‐8 assay and flow cytometry, respectively. ***p < 0.001. ^#^ *p* < 0.05, ^##^ *p* < 0.01, ^###^ *p* < 0.001 versus LPS + PBS.

Next, we examined the effects of exosome uptake on BV2 cell viability and apoptosis. LPS treatment significantly reduced BV2 cell viability at 48 h (Figure 2a, *p* < 0.001), whereas internalization of exosomes from UCMSCs, BMMSCs, or DPMSCs partially restored viability. Among them, DPMSCs‐Exo exerted the most pronounced effect (*p* < 0.001). Consistently, LPS‐induced apoptosis was most effectively attenuated by DPMSCs‐Exo (Figure 2b, *p* < 0.001).

Based on these results, DPMSCs and DPMSCs‐Exo were selected for subsequent experiments.

### 3.2 Proteomics Analysis of DPMSCs‐Exo

**FIGURE 3:**
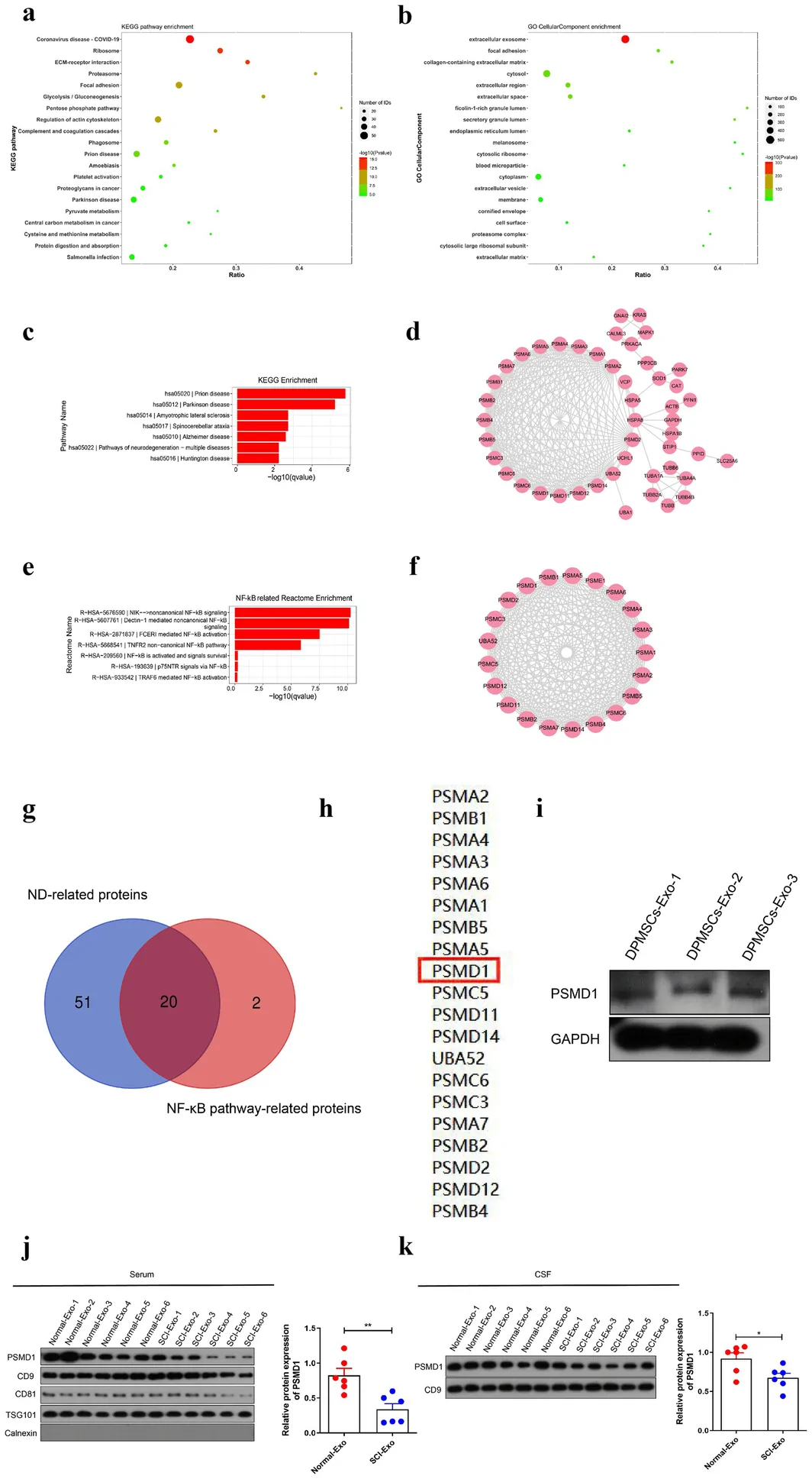
Proteomics analysis of DPMSCs‐Exo. (a) KEGG and (b) GO_CC enrichment analyses of DPMSCs‐Exo. (c) Screening of entries related to neurodegenerative diseases among the 727 identified proteins. (d) Neurodegenerative disease‐related proteins from the 727 proteins were visualized using a PPI network. (e) Reactome enrichment analysis showing significant enrichment of the NF‐κB pathway. (f) NF‐κB pathway‐related proteins were screened for PPI analysis and visualized in Cytoscape. (g) Venn diagram demonstrating the intersection between ND‐related proteins and NF‐κB pathway‐related proteins. (h) Twenty overlapping proteins were obtained. (i) Among these, only PSMD1 was detected in DPMSCs‐Exo. (j,k) PSMD1 levels in SCI‐Exo and Normal‐Exo from (j) serum and (k) CSF. *p < 0.05, **p < 0.01.

LC–MS/MS proteomic analysis identified 727 proteins in DPMSCs‐Exo. KEGG pathway enrichment analysis revealed significant representation of neurological disease‐associated pathways, including prion disease and Parkinson's disease (Figure 3a). GO_CC analysis demonstrated enrichment in categories such as extracellular vesicle, extracellular region/space, collagen‐containing extracellular matrix, extracellular exosome, and extracellular matrix (Figure 3b).

To further investigate the relevance of these proteins to neurodegenerative conditions, all 727 identified proteins were screened for entries associated with neurodegenerative diseases, and the results were visualized (Figure 3c). These disease‐related proteins were subsequently extracted and analyzed for PPI networks (Figure 3d). Reactome enrichment analysis further highlighted significant involvement of the NF‐κB signaling pathway (Figure 3e).

Proteins involved in the NF‐κB pathway were then isolated for additional PPI analysis and visualized using Cytoscape (Figure 3f). By intersecting the sets of neurodegenerative disease‐related proteins and those associated with the NF‐κB pathway, 20 overlapping proteins were identified. These included PSMA2, PSMB1, PSMA4, PSMA3, PSMA6, PSMA1, PSMB5, PSMA5, PSMD1, PSMC5, PSMD11, PSMD14, PSMC6, PSMC3, PSMA7, PSMB2, PSMD2, PSMD12, PSMB4 (all proteasome subunits), and UBA52 (Figure 3g,h).

Among these overlapping proteins, PSMD1 was previously reported to be closely associated with the progression of several neurodegenerative diseases [32, 33]. In our study, PSMD1 was detected in DPMSCs‐Exo (Figure 3i). Notably, PSMD1 levels were significantly decreased in SCI‐Exo compared with those from healthy controls (normal‐Exo) (Figure 3j,k; serum, *p* < 0.01; CSF, *p* < 0.05), suggesting a critical role of exosomal PSMD1 in the pathogenesis of SCI. Based on these findings, PSMD1 was selected for subsequent investigations.

### 3.3 DPMSC‐Derived Exosomal PSMD1 Promotes M2 Polarization and Suppresses Inflammation via the JAK2/STAT3 Pathway in LPS‐Induced BV2 Cells

**FIGURE 4:**
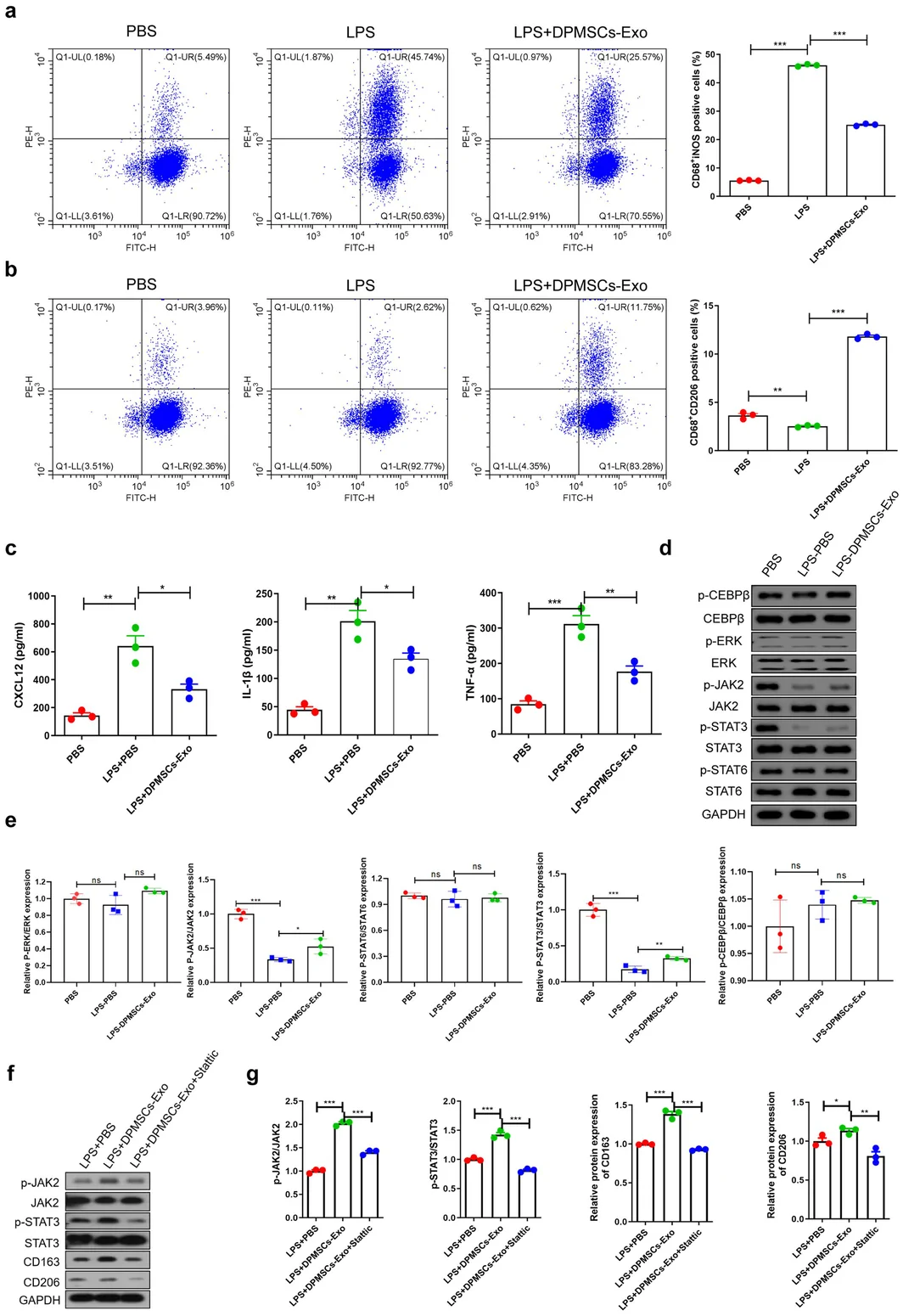
DPMSCs‐derived exosomes promote M2 polarization and inhibit inflammation via the JAK2/STAT3 pathway. Following DPMSCs‐Exo uptake, (a) percentages of CD68^+^iNOS^+^ and (b) CD68^+^CD206^+^ BV2 cells were determined by flow cytometry. (c) Levels of pro‐inflammatory cytokines (CXCL12, IL‐1β, and TNF‐α) were quantified by ELISA. (d,e) JAK2/STAT3‐, ERK‐, and CEBPβ‐related proteins were measured by western blotting. (f,g) Following treatment with stattic, the expression of p‐JAK2, JAK2, p‐STAT3, STAT3, and M2 polarization markers (CD206, CD163) was analyzed by western blotting. *p < 0.05, **p < 0.01, ***p < 0.001. ns: not significant.

Flow cytometry showed that LPS treatment markedly increased the proportion of CD68^+^iNOS^+^ BV2 cells (Figure 4a, *p* < 0.001) while reducing the proportion of CD68^+^CD206^+^ cells (Figure 4b, *p* < 0.01), suggesting suppression of the M2 phenotype. Internalization of DPMSCs‐Exo reversed these effects, significantly reducing the M1‐associated CD68^+^iNOS^+^ population while increasing CD68^+^CD206^+^ M2‐like cells (Figure 4a,b, *p* < 0.001), suggesting a shift from a pro‐inflammatory to an anti‐inflammatory microglial state. Consistently, LPS stimulation significantly elevated the secretion of pro‐inflammatory cytokines, including CXCL12, IL‐1β, and TNF‐α (Figure 4c, *p* < 0.01), which are characteristic downstream mediators of M1 activation. Treatment with DPMSCs‐Exo notably suppressed these M1‐associated inflammatory cytokines (*p* < 0.05), further confirming its inhibitory effect on the pro‐inflammatory microglial phenotype. We next investigated the effects of DPMSC‐derived exosomes on M2 polarization‐related signaling pathways, including JAK2/STAT3, ERK, and CEBPβ (Figure 4d,e). Compared with the PBS control group, LPS stimulation significantly altered pathway activation (p‐JAK2/JAK2, *p* < 0.001; p‐STAT3/STAT3, *p* < 0.001), while treatment with DPMSCs‐Exo markedly increased the p‐JAK2/JAK2 (*p* < 0.05) and p‐STAT3/STAT3 ratios (*p* < 0.01). In contrast, no significant differences were observed in p‐STAT6/STAT6, p‐ERK/ERK, or p‐CEBPβ/CEBPβ among groups. To confirm the involvement of the JAK2/STAT3 pathway, LPS‐induced BV2 cells were treated with 10 μM stattic, a STAT3 inhibitor. As shown in Figure 4f,g, stattic abolished the DPMSCs‐Exo‐induced increases in p‐JAK2/JAK2, p‐STAT3/STAT3, and the M2 markers CD163 and CD206 (all *p* < 0.01), confirming that DPMSCs‐Exo simultaneously suppressed M1‐associated inflammatory responses and promoted M2‐associated signaling via the JAK2/STAT3 pathway.

**FIGURE 5:**
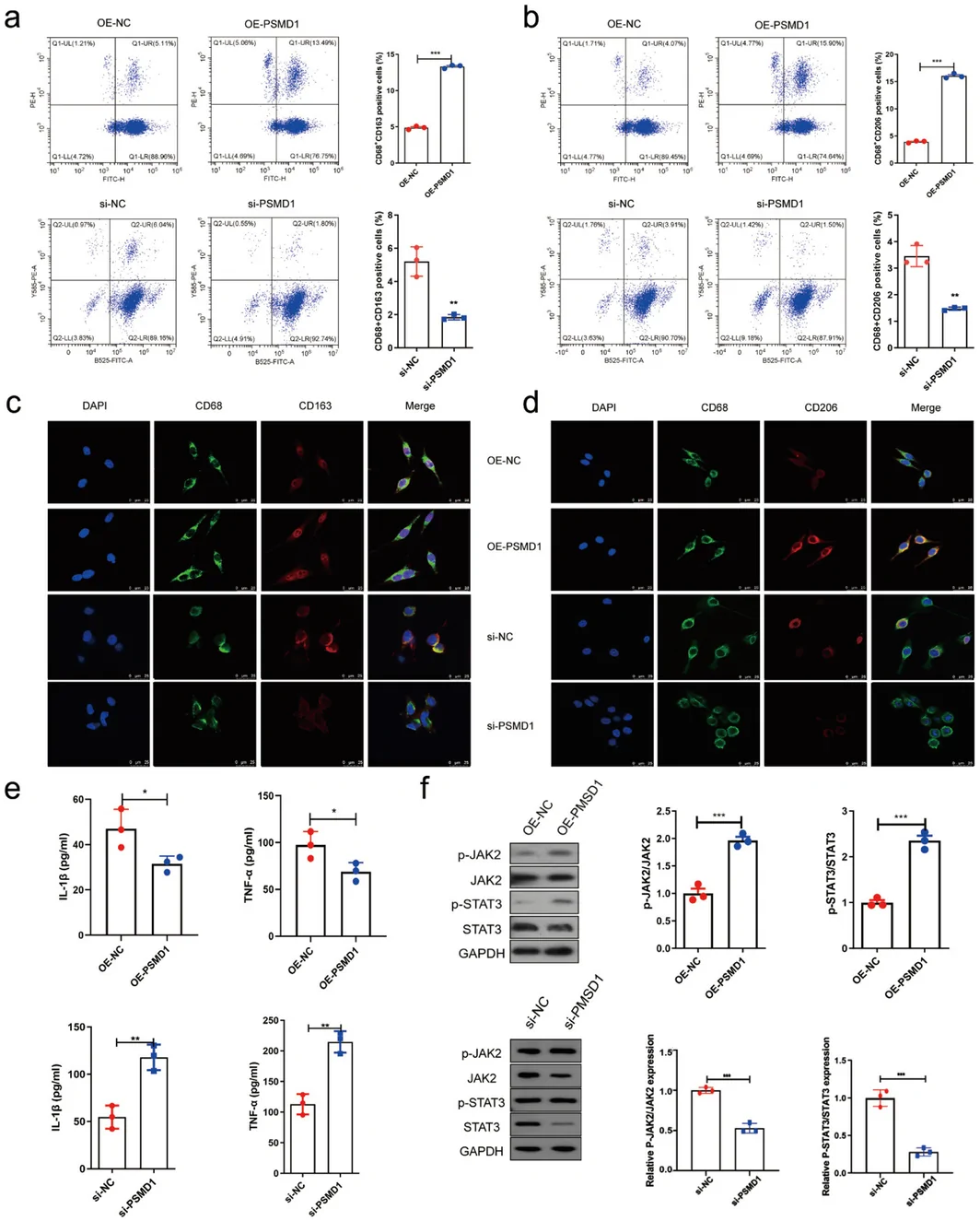
PSMD1 regulates M2 polarization and inflammatory responses in LPS‐induced BV2 cells. (a,b) Flow cytometry was used to determine the percentages of CD68^+^CD163^+^ and CD68^+^CD206^+^ BV2 cells after transfection with OE‐PSMD1, OE‐NC, si‐PSMD1, or si‐NC. (c,d) Immunofluorescence staining of myeloid marker CD68 and M2 polarization markers CD163 and CD206 in BV2 cells under indicated treatments. (e) ELISA measurement of IL‐1β and TNF‐α levels in LPS‐induced BV2 cells following PSMD1 overexpression or knockdown. (f) Western blot analysis of p‐JAK2, JAK2, p‐STAT3, and STAT3. *p < 0.05, ***p < 0.001.

To further determine the role of PSMD1 in M2 polarization and inflammation, LPS‐induced BV2 cells were transfected with either OE‐PSMD1 or si‐PSMD1‐1. As shown in Figure 5a,b, OE‐PSMD1 significantly increased the proportions of CD68^+^CD163^+^ and CD68^+^CD206^+^ BV2 cells (*p* < 0.001), while PSMD1 knockdown showed the opposite effect (*p* < 0.01). Immunofluorescence staining further confirmed that OE‐PSMD1 enhanced, while si‐PSMD1 reduced, the expression of myeloid marker CD68 and M2 polarization markers CD163 and CD206 (Figure 5c,d). Consistently, OE‐PSMD1 decreased IL‐1β and TNF‐α levels (both *p* < 0.05), whereas si‐PSMD1 increased their expression (both *p* < 0.01) in LPS‐induced BV2 cells in comparison with those transfected with their corresponding controls (Figure 5e). Additionally, transfection of OE‐PSMD1 activated the JAK2/STAT3 pathway, while PSMD1 knockdown inhibited pathway activation in BV2 cells (Figure 5f, all *p* < 0.001).

**FIGURE 6:**
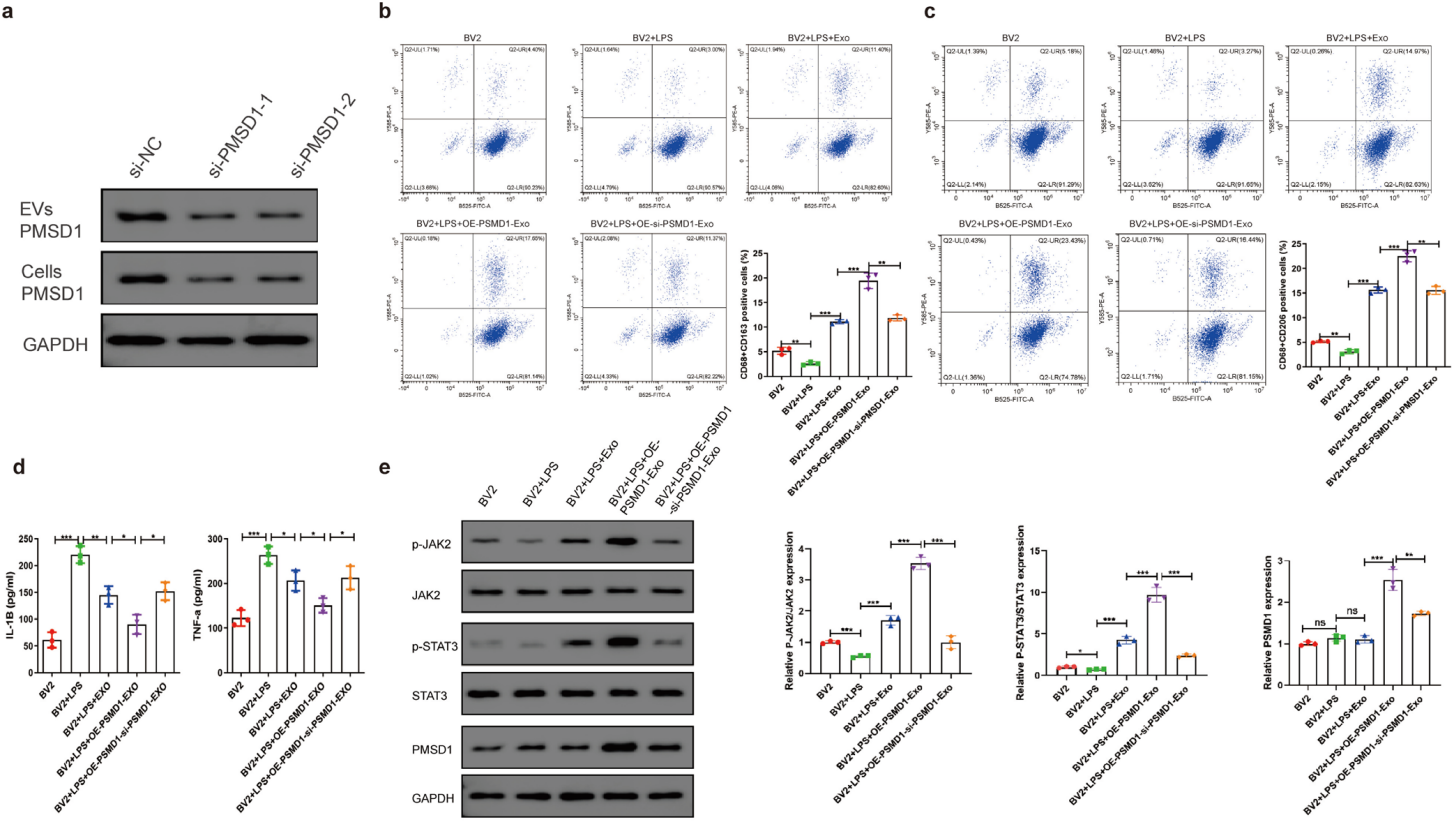
PSMD1 depletion attenuates the immunomodulatory effects of DPMSC‐derived exosomes. (a) Protein levels of PSMD1 in DPMSCs and DPMSC‐derived exosomes following transfection with si‐NC, si‐PSMD1‐1, or si‐PSMD1‐2. (b,c) Flow cytometric analysis of CD68^+^CD163^+^ and CD68^+^CD206^+^ BV2 cells following treatment with DPMSCs‐Exo, DPMSCs‐OE‐PSMD1‐Exo, or DPMSCs‐OE‐PSMD1‐si‐PSMD1‐Exo. (d) ELISA measurement of IL‐1β and TNF‐α levels in BV2 cells. (e) Western blot analysis of p‐JAK2, JAK2, p‐STAT3, STAT3, and PSMD1 protein expression in BV2 cells.

To further verify that PSMD1 is a functional exosomal cargo, DPMSCs were transfected with two independent PSMD1‐targeting siRNAs (si‐PSMD1‐1 and si‐PSMD1‐2). Western blot analysis confirmed that PSMD1 expression was significantly reduced in both DPMSCs and their derived exosomes compared with the si‐NC group (Figure 6a). Rescue experiments were subsequently performed in LPS‐stimulated BV2 cells. Flow cytometric analysis showed that DPMSCs‐OE‐PSMD1‐Exo significantly increased the proportions of CD68^+^CD163^+^ and CD68^+^CD206^+^ cells compared with DPMSCs‐Exo, whereas knockdown of PSMD1 in DPMSCs partially abolished this effect (Figure 6b,c). Consistently, DPMSCs‐OE‐PSMD1‐Exo markedly reduced IL‐1β and TNF‐α levels, while PSMD1 depletion attenuated the anti‐inflammatory effects of the exosomes (Figure 6d). Furthermore, DPMSCs‐OE‐PSMD1‐Exo enhanced PSMD1 expression and activation of the JAK2/STAT3 pathway in BV2 cells, as evidenced by increased p‐JAK2/JAK2 and p‐STAT3/STAT3 ratios. These effects were partially reversed following PSMD1 knockdown (Figure 6e). Together, these findings further support PSMD1 as a critical mediator of the immunomodulatory activity of DPMSC‐derived exosomes.

**FIGURE 7:**
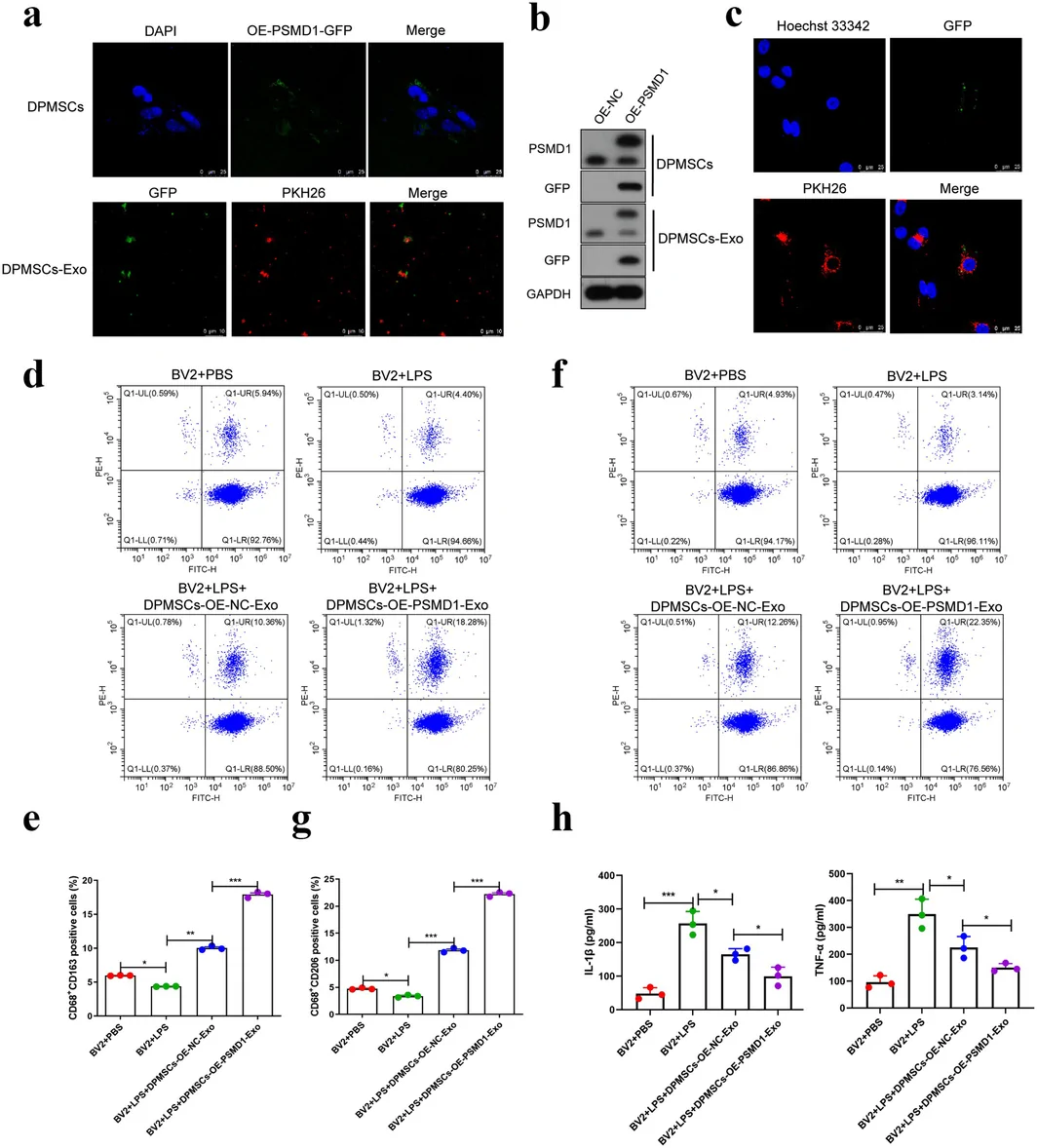
DPMSCs‐derived exosomal PSMD1 promotes M2 polarization and inhibits inflammation in LPS‐induced BV2 cells. (a) PSMD1 expression in DPMSCs and DPMSCs‐Exo was detected by immunofluorescence staining. (b) Protein levels of PSMD1 in DPMSCs and DPMSCs‐Exo were measured by western blotting. (c) Internalization of DPMSCs‐OE‐PSMD1‐Exo by LPS‐induced BV2 cells. (d,g) Flow cytometry analysis of CD68^+^CD163^+^ and CD68^+^CD206^+^ cells following exosome uptake. (h) Levels of IL‐1β and TNF‐α determined by ELISA. *p < 0.05, **p < 0.01, ***p < 0.001.

A DPMSC cell line stably expressing PSMD1‐GFP was generated to investigate exosome‐mediated delivery of PSMD1. PSMD1 expression was confirmed in both DPMSCs and their derived exosomes (Figure 7a,b). These exosomes were successfully internalized by LPS‐induced BV2 cells (Figure 7c). Compared to DPMSC‐OE‐NC‐Exo, DPMSC‐OE‐PSMD1‐Exo exerted significantly stronger effects in promoting M2 polarization and suppressing inflammation, as indicated by increased numbers of CD68^+^CD163^+^ and CD68^+^CD206^+^ cells (Figure 7d–g, *p* < 0.01). Moreover, the LPS‐induced increase in IL‐1β and TNF‐α levels in BV2 cells was partially reduced by both DPMSC‐OE‐NC‐Exo and DPMSC‐OE‐PSMD1‐Exo, with a more pronounced effect observed in the PSMD1‐expressing group (Figure 7h, *p* < 0.05).

### 3.4 DPMSCs‐Derived Exosomal PSMD1 Indirectly Promotes NE‐4C Neural Stem Cell Survival via BV2 M2 Polarization

**FIGURE 8:**
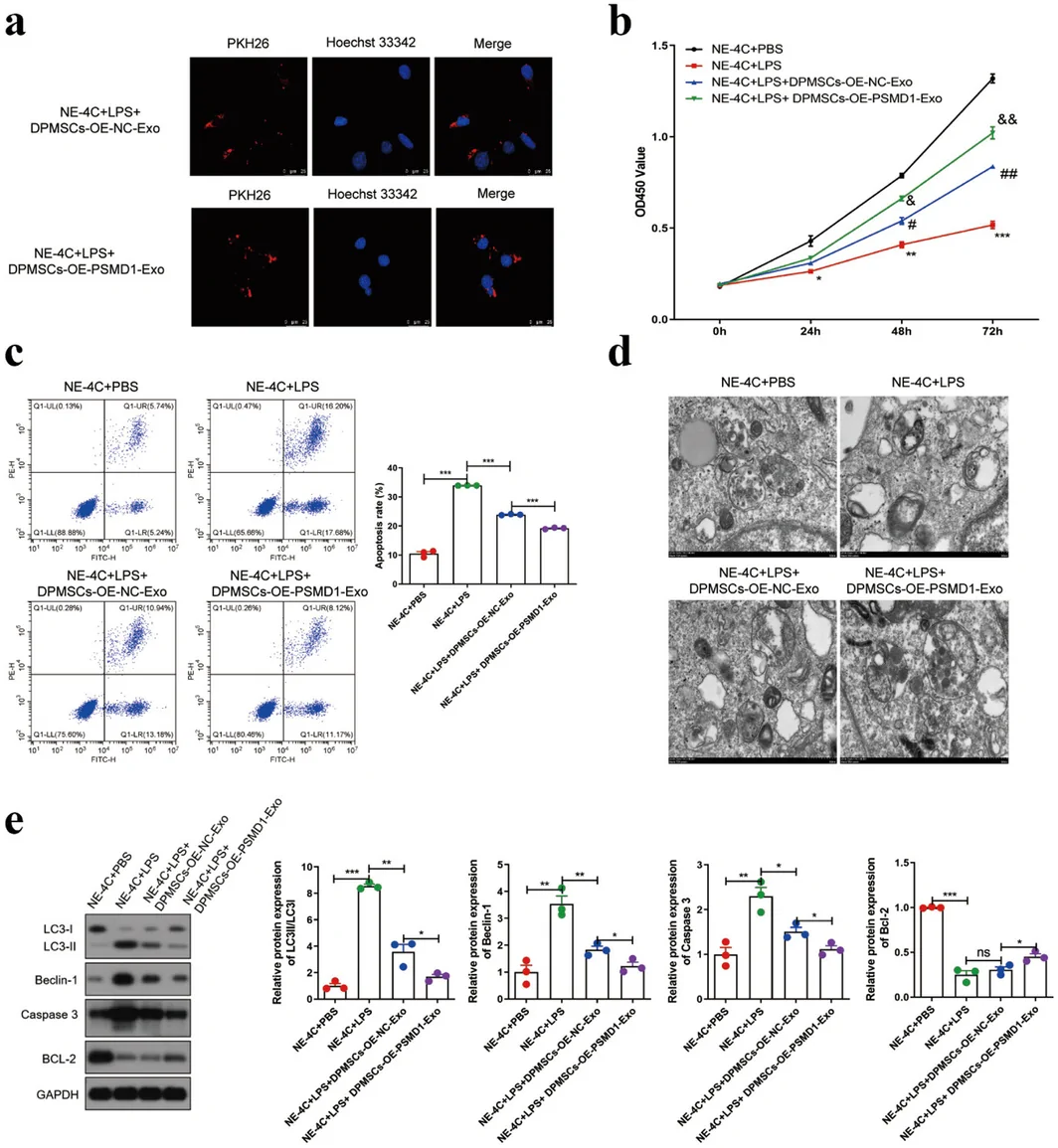
DPMSCs‐derived exosomal PSMD1 reduces apoptosis and autophagy in LPS‐induced NE‐4C cells. (a) Internalization of DPMSCs‐OE‐NC‐Exo or DPMSCs‐OE‐PSMD1‐Exo by NE‐4C cells. Following DPMSCs‐OE‐PSMD1‐Exo uptake, the (b) viability and (c) apoptosis of NE‐4C cells were assessed by CCK‐8 assay and flow cytometry, respectively. (d) Autophagosomes in NE‐4C cells were examined via TEM. (e) Autophagy‐ and apoptosis‐related proteins were detected by western blotting. *p < 0.05, **p < 0.01, ***p < 0.001. ^#^ *p* < 0.05, ^##^ *p* < 0.01 vs. NE‐4C + LPS. ^&^ *p* < 0.05, ^&&^ *p* < 0.01 vs. NE‐4C + LPS + DPMSCs‐OE‐NC‐Exo. ns: not significant.

Given the essential role of neural stem cells in axonal regeneration following SCI [40], we evaluated the effects of PSMD1‐containing DPMSCs‐Exo on NE‐4C cells. These exosomes were internalized by NE‐4C cells (Figure 8a). LPS treatment significantly decreased NE‐4C cell viability (Figure 8b, *p* < 0.05) and increased apoptosis (Figure 8c, *p* < 0.001). Treatment with either DPMSC‐OE‐NC‐Exo or DPMSC‐OE‐PSMD1‐Exo markedly improved viability and reduced apoptosis (*p* < 0.05), with the PSMD1‐Exo showing greater efficacy.

TEM revealed increased autophagosome formation in NE‐4C cells after LPS exposure, which was mitigated by internalization of DPMSC‐OE‐NC‐Exo or DPMSC‐OE‐PSMD1‐Exo (Figure 8d). Western blotting confirmed that both exosome treatments downregulated autophagy markers (i.e., Beclin‐1 and LC3II) and the pro‐apoptotic protein caspase‐3, while increasing the expression of the anti‐apoptotic protein Bcl‐2 in NE‐4C cells (Figure 8e, all *p* < 0.05).

**FIGURE 9:**
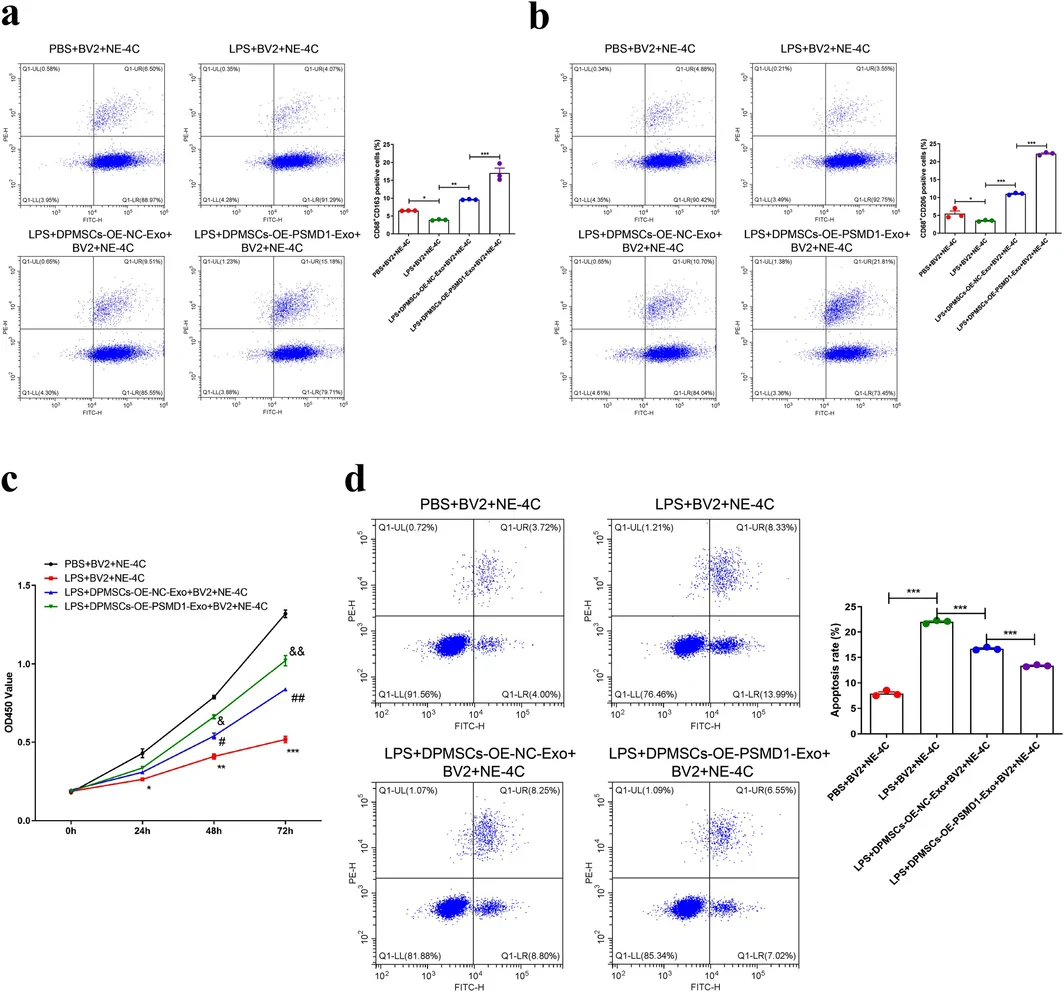
DPMSCs‐derived exosomal PSMD1 indirectly affects NE‐4C cells by promoting M2 polarization of BV2 cells. A co‐culture system was established to determine whether PSMD1‐containing DPMSCs‐Exo directly affect neural stem cells or indirectly through BV2 cells. (a,b) Flow cytometry analysis of CD68^+^CD163^+^ and CD68^+^CD206^+^ BV2 cells. The (c) viability and (d) apoptosis of NE‐4C cells were assessed using a CCK‐8 assay and flow cytometry, respectively. *p < 0.05, **p < 0.01, ***p < 0.001. ^#^ *p* < 0.05, ^##^ *p* < 0.01 vs. LPS + BV2 + NE‐4C. ^&^ *p* < 0.05, ^&&^ *p* < 0.01 vs. LPS + DPMSCs‐OE‐NC‐Exo + BV2 + NE‐4C.

To determine whether the observed effects on NE‐4C cells were direct or mediated through BV2 microglia, a BV2–NE‐4C co‐culture system was established. In the presence of NE‐4C cells, treatment with DPMSC‐derived exosomes increased the proportions of CD68^+^CD163^+^ and CD68^+^CD206^+^ BV2 cells (Figure 9a,b, *p* < 0.01), with PSMD1‐Exo exerting a significantly stronger effect (*p* < 0.001).

Moreover, NE‐4C cell viability was significantly enhanced when co‐cultured with BV2 cells treated with PSMD1‐containing exosomes (Figure 9c, *p* < 0.05), while apoptosis was markedly reduced (Figure 9d, *p* < 0.001). These findings suggest that PSMD1‐containing DPMSCs‐Exo promote NE‐4C cell survival indirectly by enhancing BV2 M2 polarization.

### 3.5 DPMSC‐Derived Exosomal PSMD1 Ameliorates SCI in a Mouse Model

**FIGURE 10:**
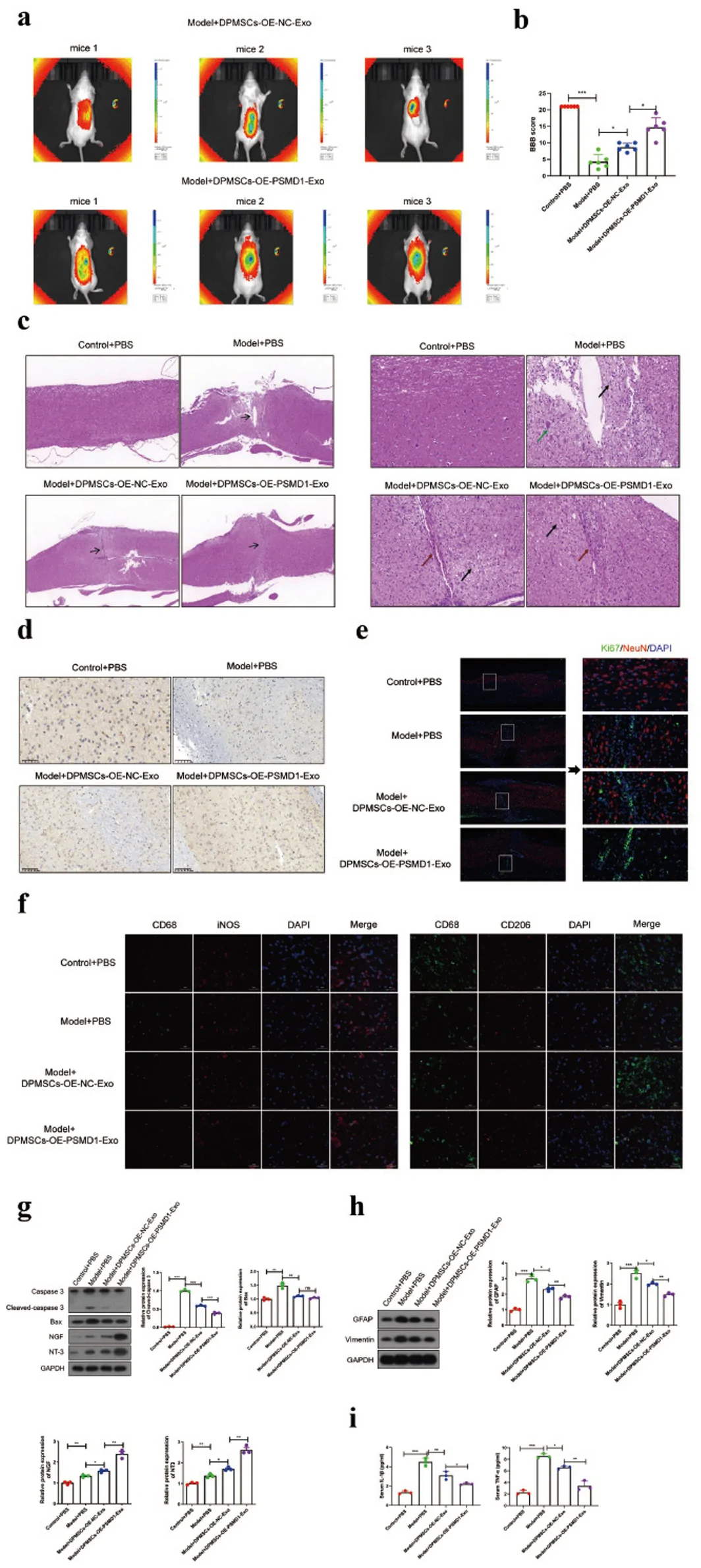
DPMSCs‐derived exosomal PSMD1 ameliorates SCI in a mouse model. (a) Representative NIRF images of the spinal cord from mice injected with DiR‐labeled DPMSCs‐OE‐NC‐Exo or DPMSCs‐OE‐PSMD1‐Exo. (b) Locomotor function was assessed using the BBB scoring system. (c) Histopathologic examination of spinal cord tissues. Black arrow: bubble‐like structures; green arrow: inflammatory cell infiltration; brown arrow: nerve fiber degeneration. (d) Immunohistochemical detection of PSMD1 expression. (e) Ki67 immunofluorescence staining to evaluate regenerative activity after SCI. (f) Immunofluorescence staining of iNOS‐ and CD206‐positive cells in spinal cord tissues. (g) Western blot analysis of apoptosis‐related proteins (cleaved caspase‐3, Bax) and neurotrophins (NGF and NT‐3). (h) Western blot analysis of activated astrocyte markers. (i) Serum IL‐1β and TNF‐α levels were determined by ELISA. *p < 0.05, **p < 0.01, ***p < 0.001. ns: Not significant.

In vivo NIRF imaging of DiR‐labeled exosomes demonstrated their successful localization to the spinal cord in mice treated with DPMSCs‐OE‐NC or DPMSCs‐OE‐PSMD1 (Figure 10a). Mice subjected to SCI exhibited a sharp decline in BBB locomotor scores (Figure 10b, *p* < 0.001). Notably, injection of PSMD1‐enriched exosomes significantly improved locomotor function compared with the controls (*p* < 0.05).

Histological analysis detected substantial tissue hyperplasia, severe nerve fiber degeneration, numerous vacuole‐like structures in the white matter, and scattered inflammatory cell infiltration in the gray matter of the Model + PBS group (Figure 10c). In contrast, the Model + DPMSCs‐OE‐NC‐Exo group exhibited only mild tissue thickening, limited nerve fiber degeneration, fewer vacuole‐like structures, and reduced inflammatory infiltration.

These pathological abnormalities were markedly alleviated in the Model + DPMSCs‐OE‐PSMD1‐Exo group. Immunohistochemical staining demonstrated that PSMD1 expression, which was downregulated in the Model + PBS group, was restored following administration of PSMD1‐containing DPMSCs‐Exo (Figure 10d).

Additionally, Ki67/NeuN immunofluorescence staining revealed a substantial number of Ki67‐positive cells at the injury site in mice treated with DPMSCs‐OE‐PSMD1‐Exo (Figure 10e), indicating enhanced regenerative activity in the damaged spinal cord tissue.

Immunofluorescence analysis further showed strong iNOS fluorescence, a marker of M1 polarization, in the Model + PBS group. This signal was reduced in both the Model + DPMSCs‐OE‐NC‐Exo and Model + DPMSCs‐OE‐PSMD1‐Exo groups (Figure 10f). In contrast, staining for the M2 polarization marker CD206 displayed an opposite trend, with the highest fluorescence intensity observed in the Model + DPMSCs‐OE‐PSMD1‐Exo group (Figure 10f), suggesting enhanced anti‐inflammatory and reparative polarization.

Astrocyte activation is known to contribute to SCI progression, whereas its inhibition has been shown to mitigate SCI pathology [41]. Western blot analysis revealed that the pro‐apoptotic proteins, cleaved caspase‐3 and Bax, were markedly upregulated in the Model + PBS group (Figure 10g, *p* < 0.01), but their expression was markedly reduced following treatment with either DPMSCs‐OE‐NC‐Exo or DPMSCs‐OE‐PSMD1‐Exo (*p* < 0.01). These results suggest that DPMSCs‐OE‐PSMD1‐Exo can reduce apoptosis in injured spinal tissue.

Interestingly, compared with the Control + PBS group, neurotrophic factors NGF and NT‐3 were significantly upregulated in the Model + PBS group (Figure 10g, *p* < 0.01), possibly reflecting spontaneous recovery following SCI. These neurotrophin levels were further increased upon treatment with DPMSCs‐OE‐NC‐Exo or DPMSCs‐OE‐PSMD1‐Exo (*p* < 0.05).

Because GFAP and vimentin are established markers of astrocyte activation [42], their expression levels were evaluated. Both markers were significantly upregulated in the Model + PBS group (Figure 10h, *p* < 0.001), but their expression was substantially reduced following administration of DPMSCs‐OE‐PSMD1‐Exo (*p* < 0.01).

Finally, serum levels of the inflammatory cytokines IL‐1β and TNF‐α were markedly elevated in the Model + PBS group (Figure 10i, *p* < 0.001), whereas both cytokines were significantly decreased following exosome treatment (*p* < 0.05).

## 4 Discussion

This study demonstrates that PSMD1‐enriched DPMSC‐derived exosomes exert potent anti‐inflammatory and cytoprotective effects on LPS‐stimulated BV2 microglia. Specifically, exosomal PSMD1 not only promoted a phenotypic shift of microglia toward the M2 state, as evidenced by increased CD206 expression, but also significantly suppressed the M1‐like pro‐inflammatory phenotype, including reduced CD68^+^iNOS^+^ cell populations and decreased secretion of M1‐associated cytokines such as IL‐1β and TNF‐α through activation of the JAK2/STAT3 pathway. This polarization of microglia enhanced NE‐4C neural stem cell survival and reduced both apoptosis and autophagy under inflammatory conditions.

Consistent with the in vitro findings, administration of PSMD1‐containing exosomes in a mouse SCI model was associated with improved locomotor function, promoted spinal tissue regeneration at the injury site, attenuated neuroinflammation and astrocyte activation, and enhanced M2 microglial polarization. These findings collectively identify exosomal PSMD1 as a potential mediator of DPMSC‐induced neuroprotection and functional recovery after SCI. It is worth noting that NeuN expression reflects mature neuronal status and may not directly correlate with early regenerative activity after SCI. The observed reduction in NeuN signal may represent transient neuronal stress or phenotypic modulation rather than a lack of regenerative response, particularly in the presence of increased proliferative activity indicated by Ki67 staining.

Microglia‐mediated inflammation is a key driver of secondary injury following SCI [43]. After the initial insult, infiltrating macrophages and activated microglia rapidly accumulate at the lesion site, generating a sustained inflammatory response that exacerbates neuronal loss and tissue damage [44]. Immune cells exhibit high plasticity and can adopt either an anti‐inflammatory, tissue‐repairing M2 phenotype or a pro‐inflammatory M1 phenotype in response to the local injury microenvironment [45]. An imbalance toward M1 polarization is a hallmark of SCI, characterized by excessive pro‐inflammatory cytokine production and persistent inflammatory signaling, which collectively hinder axonal regeneration and functional recovery [46]. Previous evidence has shown that MSC‐derived extracellular vesicles can modulate microglial and macrophage polarization by delivering specific bioactive cargo. For instance, exosomes enriched in ubiquitin‐specific protease 29, derived from melatonin‐preconditioned MSCs, have been reported to promote functional recovery following traumatic SCI [47]. Additionally, MSC‐derived exosomal microRNAs exert anti‐inflammatory effects in various SCI animal models [48]. Consistently, we found that DPMSC‐derived exosomal PSMD1 effectively promoted microglial polarization toward the M2 phenotype, accompanied by enhanced anti‐inflammatory signaling and reduced pro‐inflammatory cytokine production. These results highlight the critical role of microglial polarization in SCI pathophysiology and identify exosomal PSMD1 as a novel regulator of microglia‐mediated inflammation.

The JAK2/STAT3 signaling pathway is a well‐established regulator of microglial polarization and neuroimmune homeostasis [35]. Upon activation, phosphorylated STAT3 translocates to the nucleus, where it facilitates the transcription of M2‐associated genes such as *IL‐10* and *CD206*, while simultaneously repressing pro‐inflammatory mediators including *TNF‐α*, *IL‐1β*, and *iNOS*. This dual action facilitates tissue repair in central nervous system injury [49, 50]. In SCI, sustained activation of the JAK2/STAT3 signaling pathway in microglia is associated with improved debris clearance, resolution of inflammation, and support for axonal regeneration [51]. Exosome‐mediated modulation of the JAK2/STAT3 pathway has also been reported in SCI. For example, exosomes derived from endothelial progenitor cells can promote anti‐inflammatory macrophage polarization via JAK2/STAT3 signaling, thereby enhancing functional recovery [52]. Mechanistically, proteasomal subunits, including PSMD1, have been implicated in the regulation of STAT activation and downstream inflammatory pathways [53, 54]. In line with these findings, our data demonstrated that DPMSC‐derived exosomal PSMD1 activated JAK2/STAT3 signaling in BV2 microglia, thereby promoting M2 polarization and suppressing pro‐inflammatory responses. These results build upon previous observations by identifying PSMD1 as a functional exosomal cargo that connects proteostasis to microglial programming in the context of SCI.

Effective crosstalk between microglia and neural stem cells is increasingly considered a key determinant of neural repair following SCI [55], as microglia play an active role in regulating neuronal survival, axonal preservation, and the behavior of other glial cells within the injured spinal cord microenvironment [56]. M2‐polarized microglia and their secreted exosomes promote neuronal survival and axonal integrity, inhibit reactive astrocyte activation, and ultimately support tissue repair and motor function recovery in both in vitro and rodent models of SCI [57, 58]. Furthermore, MSC‐derived exosomes can modulate the inflammatory microenvironment and, in turn, enhance neural cell resilience. Exosomal cargo, including proteins, mRNAs, and microRNAs, from hypoxia‐preconditioned MSCs have been reported to induce a phenotypic shift from M1 to M2 microglia, thereby reducing inflammation and improving neural outcomes in SCI models [59].

Consistent with these findings, our study demonstrated that DPMSC‐derived exosomal PSMD1 promoted M2 microglial polarization, which, in turn, enhanced neural stem cell survival and suppressed both apoptosis and autophagy in NE‐4C cells.

Collectively, these results identify PSMD1 not only as a potential regulator of microglial immune phenotype but also as a mediator of indirect neuroprotection, contributing to the repair process following SCI.

Several limitations of this study should be acknowledged. First, although our findings demonstrate the short‐term neuroprotective and anti‐inflammatory effects of DPMSC‐derived exosomal PSMD1, long‐term studies are warranted to evaluate the durability of functional recovery and spinal tissue regeneration following SCI. Second, exosomes in this study were isolated using a precipitation‐based method, which may co‐isolate non‐exosomal proteins or vesicular contaminants and thus could introduce potential bias in attributing biological effects exclusively to exosomes. In addition, the composition of exosomal cargo may vary between DPMSC donors, potentially introducing variability in therapeutic efficacy. Therefore, standardization of exosome production and characterization will be critical for future translational applications. Third, while patient‐derived samples were included, the sample size was relatively small, and limited clinical information was available, including injury severity, spinal level, and sampling time after injury. Therefore, these data should be interpreted cautiously. Larger cohorts with more comprehensive clinical characterization will be required in future studies. Likewise, the in vivo experiments were conducted with a limited sample size and a relatively short observation period of 1 week, which restricts the assessment of long‐term functional recovery and sustained histological outcomes. Fourth, although PSMD1 overexpression and knockdown experiments suggest a functional role in regulating microglial polarization, these approaches alone are not sufficient to definitively establish PSMD1 as the key active exosomal component. Additional mechanistic validation, such as a dual‐luciferase reporter assay, will be necessary to further confirm its causal role. Fifth, while we identified activation of the JAK2/STAT3 signaling pathway as a key mechanism by which exosomal PSMD1 promotes M2 microglial polarization, exosomes are complex vesicles that carry a diverse array of biomolecules (e.g., lipids, RNAs, and additional proteins) that may also contribute to their neuroprotective effects. Additionally, the interaction between M2‐polarized microglia and neural stem cells is complex. Prior studies suggest that neural stem cell activation may precede or act synergistically with M2 polarization to promote recovery [60]. Further investigations are warranted to explore the temporal and functional relationships between these cell populations in SCI repair.

## 5 Conclusion

This study demonstrates that DPMSC‐derived exosomes enriched with PSMD1 exert potent neuroprotective effects in SCI by promoting M2 microglial polarization and suppressing pro‐inflammatory signaling through activation of the JAK2/STAT3 pathway. These results suggest that PSMD1 may contribute to the regulatory effects of DPMSC‐derived exosomes on microglial polarization and neuroinflammatory responses. Overall, this work provides preliminary evidence for future investigation into exosome‐mediated delivery systems and highlights the potential involvement of PSMD1 in neural repair processes following central nervous system injury.

## Author Contributions

Xiaohui Xing and Yuan Hao performed the formal analysis, investigation, methodology design, software implementation, data visualization, drafted the original manuscript, and contributed to manuscript revision. Guanghui Zhang, Xiaohui Xing, Hongfei Jia, and Chenlong Liu performed the formal analysis, methodology design, software implementation, data visualization, and manuscript revision. Xin Wang performed the manuscript revision. Tao Li, Xueyuan Li, and Yilei Xiao performed conceptualization, data curation, project administration, supervision, validation, and manuscript revision. All authors read and approved the final manuscript.

## Funding

This work was supported by the Chinese Medicine Science and Technology Project of Shandong Province of China, M‐2022123. The Key Research and Development Plan of Liaocheng City, Sha, 2023YD42. The Natural Science Foundation of Shandong Province of China, ZR2021MH303. The Taishan Scholar Project of Shandong Province of China, tsqn202103200. The Youth Scientific Research Fund of Liaocheng People's Hospital, 201910915. The Affiliated Hospital (Teaching Hospital) Research and Development Foundation of Shandong Second Medical University, 2024FYM138.

## Ethics Statement

This study was performed in line with the principles of the Declaration of Helsinki. Approval was granted by the Institutional Ethics Committee (approval no. 2024258). Written informed consent was obtained from all participants. All animal procedures were conducted in accordance with the NIH Guide for the Care and Use of Laboratory Animals and were approved by the same committee (approval no. 2024–02‐19A).

## Consent

The authors have nothing to report.

## Conflicts of Interest

The authors declare no conflicts of interest.

## Data Availability

The data that support the findings of this study are available from the corresponding author upon reasonable request.
